# Cast OFF-2: 1 week of plaster cast immobilization for non-reduced distal radius fractures—a study protocol for an implementation study

**DOI:** 10.1186/s13063-021-05889-z

**Published:** 2021-12-19

**Authors:** Emily Boersma, Erik van de Krol, Tjarda Tromp, Maria Nijhuis - van der Sanden, Michael Edwards

**Affiliations:** 1 Department of Surgery, Radboud University Medical Center, Radboud Institute for Health Sciences, route 618, P.O. Box 9101, NL-6500 HB Nijmegen, the Netherlands; 2 Department of IQ Healthcare, Radboud University Medical Center, Radboud Institute for Health Sciences, Nijmegen, the Netherlands

**Keywords:** Distal radius fracture, Plaster cast immobilization, One-week immobilization, Implementation study

## Abstract

**Background:**

The distal radius fracture (DRF) is a common fracture, with the majority of these fractures being stable. Of all diagnosed fractures, 17% is a DRF, of which a large part is extra-articular and one-third is non-displaced. There is a large variation in treatment advisements for non-reduced DRF. Four to 5 weeks of immobilization is often the usual practice. Existing evidence shows that 1 week of immobilization is safe and does not lead to an increase in secondary displacement. Additionally, shorter immobilization periods may lead to less outpatient clinic visits and less home care for elderly people and may lead to earlier return to work and other social activities. Therefore, shorter immobilization periods for non-reduced distal radius fractures may also prove to be cost-effective.

In this study, we aim to successfully implement 1 week of plaster cast immobilization for non-reduced distal radius fractures in twelve medical centers and to evaluate the functional outcome and cost-effectiveness.

**Methods:**

This study will be performed using a multicenter randomized stepped wedge design in 12 centers. We aim to include in the study 440 patients with an isolated non-reduced DRF between the age of 18 and 85 years old. The patients in the intervention group will be treated with plaster cast immobilization for 1 week. Acceptability of the study protocol, patient-reported outcomes, quality of life, complications, pain catastrophizing score, pain and patient satisfaction, and cost-effectiveness will be measured. The total follow-up will be 12 months.

**Discussion:**

The strength of this study is the combination of implementing 1 week of plaster cast immobilization for non-reduced DRF and the evaluation of functional outcome, acceptability of the study protocol, and cost-effectiveness in actual practice.

**Trial registration:**

Netherlands Trial Register NL9278. Registered on 17 February 2021

## Administrative information

**Table:** 

Title	Cast OFF-2: One week of plaster cast immobilization for non-reduced distal radius fractures. A study protocol for an implementation study.
Trial registration	Registration at Netherlands Trial Register, NL9278, registration date 17-02-2021. All items of the WHO Trial Registratiom Data Set can be found in the protocol and at the Netherlands Trial Registration website.
Protocol version	Version 2, date 15 January 2021.
Funding	The Cast OFF-2 will receive funding from an international grant from the Orthopaedic Trauma Association. The funder will not have any involvement in the design, collection, management, analysis, and interpretation of the data.
Author details	The authors EB, EK, TT, ME work at the Radboud university medical center, Radboud Institute for Health Sciences, Department of Surgery. Author MN works at the Radboud university medical center, Radboud Institute for Health Sciences, Department of IQ Healthcare, Nijmegen, the Netherlands. The Institute of Neurosciences, Universitat de Barcelona, Barcelona, Spain.
	Michael Edwards, Professor of Trauma Surgery.Radboud university medical center, Department of Surgery. Email: michael.edwards@radboudumc.nl
Role of sponsor	All authors who participated in the development of the study protocol will have a role in conducting the study, analysis, interpretation of data, writing of the report and decision to submit the report for publication. Participating hospitals will be asked to participate in data sampling and writing the report for the study and thereby participating in the analysis and interpretation of data.

## Background

The distal radius fracture (DRF) is a common fracture. A large part of all diagnosed fractures at the Emergency Department are DRFs, around 17%. One-third of these fractures are non-displaced fractures [1–3].

In recent years, there has been an increased interest in the treatment of DRFs, caused by the increasing prevalence of DRFs and improvements in minimal invasive surgical treatment techniques. However, recent literature mainly focused on treatment options for unstable distal radius fractures, for which several treatment modalities have been advocated [4, 5]. Nonetheless, little attention has been paid to the treatment of the stable DRF. To date, there are only a few studies that have investigated the duration of immobilization for non-operatively treated, stable DRFs [6]. A systematic review (2018) included 12 studies, of which a small part focused on non-reduced fractures. The authors concluded that an immobilization period of 3 weeks or less is equally effective compared to the longer immobilization period and might be associated with better functional outcome [6].

There are several guidelines in the world for DRFs [4, 7]. However, a unified treatment recommendation for non-reduced (minimal and non-displaced) DRFs does not exist. The usual care in the Netherlands consists of 3- to 5-week immobilization for these kinds of fractures. There are several studies that show that the 1 week of plaster cast treatment for a stable DRF is safe and effective [6, 8, 9]. A feasibility study including 40 patients with a non-reduced DRF, randomized in an intervention group (1 week of plaster cast treatment) or control group (4–5 weeks of plaster cast treatment) showed positive results and no secondary displacement. In addition, a trend was shown in the intervention group of having less pain, better functional outcome after 6 weeks, going back to work earlier, higher patient satisfaction, and no differences in complications. Moreover, it appeared that patients preferred a shorter immobilization period (cross-over in eight patients, seven patients went from the control group to the intervention group) [10].

Recent studies have shown that a long period of immobilization is associated to more post-traumatic pain by increasing disuse and kinesiophobia [11–13]. These studies also show that early mobilization after a period of plaster cast may reduce the incidence of post-traumatic pain including complex regional pain syndrome (CRPS).

### Rationale

There is a large variation in treatment advisements for non-reduced DRFs. Existing evidence for 1-week immobilization shows that 1 week of immobilization is safe and does not lead to more secondary displacement. However, there is a lack of evidence on functional outcome, pain and pain medication use, and time to return to activities and work after non-operative treatment for non-reduced DRF. The interest for shorter immobilization periods for these fractures is high, from both professionals as well as patients. Shorter immobilization periods may lead to less outpatient clinic visits and less home care for elderly people and may lead to earlier return to work and other social activities. Shorter immobilization periods for non-reduced DRFs may therefore prove to be cost-effective.

In this study, we aim to successfully implement 1 week of plaster cast immobilization for non-reduced DRF in twelve medical centers and to evaluate the functional outcome and cost-effectiveness for 1 week of plaster cast immobilization compared to the usual care.

## Methods

The present study is a multicenter randomized stepped wedge design with ten clusters. Twelve medical centers in the Netherlands will participate in this study, which will be combined to ten equal clusters. The objective of this study is to successfully implement 1 week of plaster cast immobilization for non-reduced DRFs in twelve medical centers and to evaluate the functional outcome, acceptability of the study protocol, and cost-effectiveness.

The usual care of 4 to 5 weeks of plaster cast (condition A) will be compared to the intervention of 1 week of plaster cast immobilization (condition B). The primary outcome of this study is the acceptability of the study protocol. The secondary outcome scores are the Patient-Rated Wrist Evaluation score (PRWE), PROMIS Pain Interference, return to activity measuring with the productivity costs questionnaire (iCPQ), pain and the use of pain medication, Quality of Life (QOL) using the EuroQol-5 dimensions 5-level questionnaire (EQ-5D-5L), Pain Catastrophizing Scale (PCS-4), patient satisfaction, complications using the complication checklist of McKay measured at 6 weeks and 3, 6, and 12 months post-injury. Additionally, a cost-effectiveness analysis will be performed using a selection of questions of the iCPQ and the medical consumption questionnaire (iMCQ).

### Hypotheses

Our hypotheses in the study are as follows:

- One week of plaster cast immobilization may successfully be implemented at the participating hospitals with a good acceptability of the study protocol rate (small number of protocol violations).
- One week of plaster cast treatment (condition B) will give a lower PRWE score (less pain and better physical function) compared to the usual care group (condition A) for non-reduced DRFs.
- One week of plaster cast treatment (condition B) will give lower pain scores with less use of pain medication compared to the usual care group (condition A) for non-reduced DRFs.
- One week of plaster cast treatment (condition B) will lead to earlier optimal functional outcome scores than the usual care group (condition A) for non-reduced DRFs.
- One week of plaster cast treatment (condition B) will be less costly compared to the usual care group (condition A) for non-reduced DRF.
- There is no difference in complications for the intervention group (condition B) versus the usual care group (condition A).

### Design

The present study is a multicenter randomized stepped wedge trial. Patients with a DRF treated non-operatively and without performance of a closed reduction will be included in the study.

This stepped wedge trial will include 10 clusters, expecting to include 4 patients per cluster per time period of 4 weeks (Table 1). Every cluster will be randomized to a step: the moment when a cluster will start with the study. A transition period of one time period of 4 weeks is added between conditions A and B. This transition period is meant for explaining the treatment and to give instructions to the hospitals and caregivers to re-organize the treatment protocols.

**Table 1:** Design Cast OFF-2, stepped wedge design

Cluster	T1	T2	T3	T4	T5	T6	T7	T8	T9	T10	T11	T12
1	A		B	B	B	B	B	B	B	B	B	B
2	A	A		B	B	B	B	B	B	B	B	B
3	A	A	A		B	B	B	B	B	B	B	B
4	A	A	A	A		B	B	B	B	B	B	B
5	A	A	A	A	A		B	B	B	B	B	B
6	A	A	A	A	A	A		B	B	B	B	B
7	A	A	A	A	A	A	A		B	B	B	B
8	A	A	A	A	A	A	A	A		B	B	B
9	A	A	A	A	A	A	A	A	A		B	B
10	A	A	A	A	A	A	A	A	A	A		B

*A*, usual care, 4–5 weeks of plaster cast treatment; *B*, 1 week of plaster cast treatment

An inclusion of 4 patients per cluster per month is expected. Before starting this study, every hospital needs to indicate the number of patients seen per month with a DRF who are treated non-operatively. Hospitals of which the expected number of patients will be less than 4 patients per month will be combined to one cluster. By adapting the study protocol based on the expected available patients, we expect that all clusters will reach sufficient inclusions.

Randomization will be performed to determine when a cluster will start with the study. The study period for every included patient will be 1 year. Follow-up of the included participants is the same for both groups: conditions A and B.

### Patient selection and methods

Patients with acute non-reduced DRFs (intra- and extra-articular DRF, non- and minimal displaced DRF) who are diagnosed at the emergency department will be included in the study. Patients need to be between the age of 18 and 85 years old, have an isolated non-reduced DRF and a good understanding of the Dutch language, and live independently.

Patients will be informed about the study at the emergency department. The treating physician will inform the patient about the study and provide them with written information about the study.

After 1 week, all patients will be seen at the hospital (plaster room outpatient clinic) for their first standard of care follow-up visit. During this visit (after 1 week at the plaster room), patients will be asked by the research team if they are willing to participate in the study. Patients need to give written informed consent for the use of their medical information and medical files and filling in questionnaires. The patients will be informed about the possibility that they will be called or emailed by the research team in case of incomplete or missing questionnaires during the 1-year follow-up.

The following inclusion and exclusion criteria will be used:

*Inclusion criteria*

- Age between 18 and 85 years
- Isolated acute distal radius fracture; intra- and extra-articular
- Closed reduction is not performed
- Non-operative treatment with cast immobilization
- Ability to perform activities of daily living (ADL) independently
- Ability to give informed consent

*Exclusion criteria*

- Multiple injured patient
- Reduction is indicated/performed
- Operative treatment
- No understanding of the Dutch language
- A patient with extra care at home; in need of a caregiver or professional for ADL
- Open fractures
- History of surgically treated wrist fracture on the currently injured side
- Estimated high risk of secondary displacement by the surgeon due to comorbidities, high risk of falling, or life circumstances

### Investigational treatment

All clusters will start simultaneous with their usual treatment: condition A. After 1 week of plaster cast, patients will be seen at the outpatient clinic, a physical examination will be performed, and a prolonged plaster cast will be provided (another 2–4 weeks).

During the second follow-up visit at the outpatient clinic, the plaster cast will be removed, the duration of immobilization with the plaster cast will be registered, a physical examination will be performed, and extra questions on function and pain will be asked. After removal of the plaster cast, information is given about the importance of using their arm and performing the exercises of the home exercise program.

When the cluster/hospital is randomized to switch treatment, all patients will be treated following condition B. Patients in condition B will have an immobilization period of 1 week. Patients will get a splint or cast at the emergency department. After 1 week, an appointment is scheduled at the outpatient clinic. Instead of a plaster cast change, the splint or cast will be removed and physical examination will be performed. After the removal of the plaster cast, information is given about the importance of using their arm and doing the exercises of the home exercise program. Four to 5 weeks post-injury, patients will have a second follow-up appointment at the outpatient clinic where a physical examination will be performed and additional questions on function and pain will be asked. When patients did not give informed consent for use of their medical files and filling in questionnaires, they will still be treated by the protocol the hospital is using at that moment. That can be condition A or B.

### Measurements

For this study, the following main study parameters are gathered: functional outcome, return to activity, cost-effectiveness, and acceptability of the study protocol.

- Patient-reported outcome (functioning and patient satisfaction): It is known that 1 week of plaster cast for a non-reduced DRF is safe. Secondary displacement of the fracture is not significantly different with longer plaster cast treatment [8, 9]. So, the most important question is whether 1-week treatment gives better functional results and higher patient satisfaction. The best measurement method to measure patient satisfaction and functional result is using a patient-reported outcome (PRO) [14]. In addition, PROs are better predictors of participation, such as the ability to return to work or perform daily activities, than more objective clinical measures.
- The Patient-Reported Wrist Evaluation (PRWE) is specifically designed for wrist and hand functioning. The PRWE is a reliable and valid measure of patient-rated pain and disability for wrist conditions [15, 16]. Furthermore, a validated Dutch version of the questionnaire is available [17]. The PRWE is a measurement with a score from 0 to 100, where 0 is no wrist function problems or pain. The minimal clinical important difference for PRWE is a difference of 11 points [18].
  The questionnaires will be sent to the patient after 6 weeks and 3, 6, and 12 months.
- Return to activity will be measured using the productivity costs questionnaire (iCPQ). Questions about return to work, leisure activities, household activities, and pain will be asked. These questions will be asked after 1 and 6 weeks, 6 months, and 1 year.
- A cost-effectiveness analysis will be performed alongside the stepped wedge trial. The Health care Institute of the Netherlands (ZIN) 2016 guideline for economic evaluations forms the basis of the analysis [19]. Healthcare consumption from inclusion to final follow-up of the patient is measured. This is in line with the measurement pathway of the clinical study. Healthcare consumption is multiplied by unit costs associated with healthcare consumption. Also, productivity-related costs seem to play an important role. Therefore, the societal perspective is the base case. A healthcare perspective is used as the scenario. These will be measured using a selection of questions of the medical consumption questionnaire (iMCQ) and the productivity costs questionnaire (iCPQ). These questions will be asked after 1 and 6 weeks, 6 months, and 1 year. The costs and for example months not working will be compared for the two different condition groups.
- Acceptability of the study protocol. Measuring the actual time of plaster cast treatment and number of patients who followed the protocol. If not following the protocol (condition A or B), reason for violation of the protocol. The answers to these questions will be gathered at the end of the study by provided information from the hospitals and questions asked to the participating physicians. All patients who gave informed consent will be checked if they follow the correct protocol. If they did not follow the protocol, information will be gathered why the protocol was not followed. Additionally, questions will be asked to the participating centers (local head of research). The questions asked are about feedback to the study, if they will implement the interventional treatment, why they will or will not implement the treatment, what changes to the treatment they would make, etc. The number of protocol violations and comments will be compared for both groups.

The following secondary parameters will be measured.

**Fig. 1:**
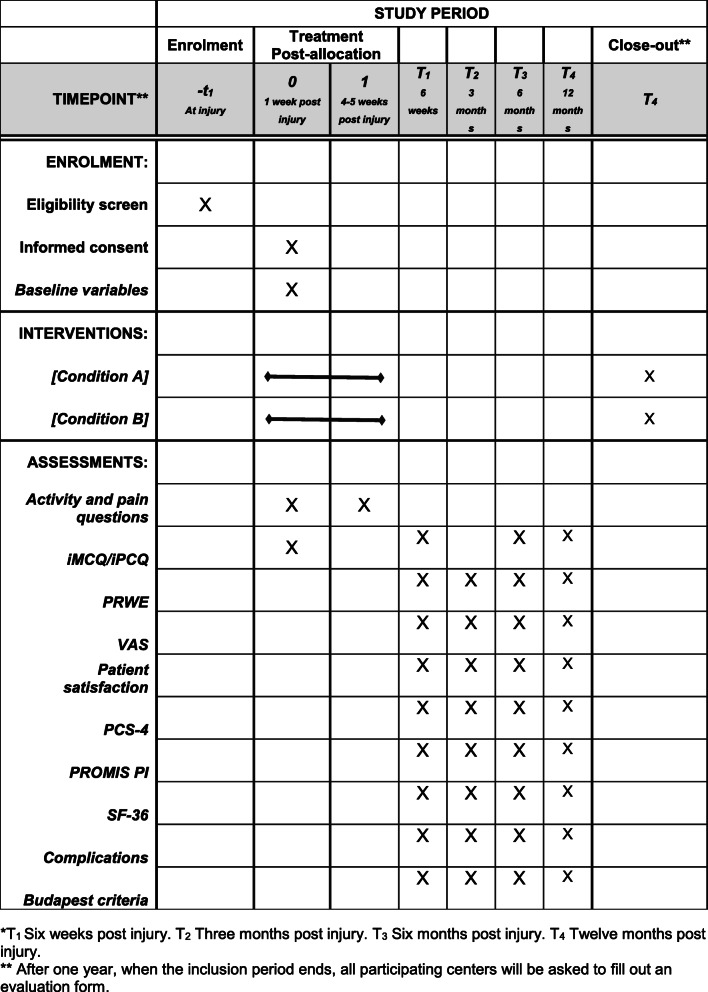
Time schedule for the included patients of the Cast OFF-2
- Comprehensive PRO measures will be gathered. This is a more generic instrument that captures aspects of health status and quality of life beyond hand functioning. The questionnaire is useful to identify the contribution to overall disability. The EuroQol-5 dimensions 5-level questionnaire (EQ-5D-5L) [23]. This questionnaire is sent after 6 weeks and 3, 6, and 12 months post-injury.
- Patient satisfaction will be additionally measured by using a 10-point ordinary scale, 0 is not satisfied with the treatment and 10 is very satisfied with the treatment. As this is an interesting question but not a validated question for wrist fractures, the minimal important clinical difference is not known. The difference between the groups will be analyzed. The questions will be asked after 6 weeks and 3, 6, and 13 months.
- A budget impact analysis will be performed from various perspectives with the budgetary framework for the care as the base case perspective (budgettair kaderzorg). Here too, the ZIN 2016 guideline serves as a starting point for the analysis. The analysis will be performed in accordance with the ISPOR Principles of Good Practice for Budget Impact Analysis [24].

- Complications. The complication checklist for DRF from McKay will be used for scoring the complications after a DRF [20]. The clinical record and questionnaire will be used to complete the checklist from McKay. The Budapest diagnostic criteria will be used to score CRPS, a complication which can occur after a DRF [21]. In addition, pain and post-traumatic pain will be scored. The visual analogue scale (VAS) will be asked, and the PROMIS questionnaire will be used for pain interference and diagnosing post-traumatic pain [11, 22]. In addition, we will measure the use of pain medication by using the iMCQ and iCPQ. Figure 1 shows the moment when these parameters are gathered.

The following other study parameters such as demographic and clinical data will be collected to measure the association with the above-mentioned outcome parameters.

- Age at inclusion
- Gender
- Side of fracture
- Side of the dominant hand
- Smoking
- Use of pain medication, if yes which pain medication and dose at inclusion
- VAS score at inclusion
- Use of vitamin C
- Work or retired and specified
- Duration not able to work or perform household activities
- Leisure activities
- Fracture classification using AO classification system
- Pain catastrophizing scale (PCS-4) will be used to measure patients’ fear for movement and the influence of psychological problems [25]
- Use of physiotherapy, asked after 1 year
- Use of home exercise program
- Comorbidities using Cumulative Illness Rating Scale (CIRS)

### Follow-up

The follow-up of the included patient will be the same for both groups, conditions A and B. All questionnaires will be sent via email. The follow-up will take place per participant/patient. The patients will be informed about the possibility that they will be called or emailed by the research team in case of incomplete or missing questionnaires during the 1-year follow-up.

Patients will be informed about the importance of finishing the questionnaires and will be called or contacted via email if they did not complete the questionnaires.

#### Outpatient clinic follow-up

The outpatient clinic follow-up will take place after 1 week and 4–5 weeks. During these visits, several questions about pain and performing activity will be asked.

#### Pain and activity

The pain catastrophizing scale will be sent 6 weeks post-injury and 3, 6, and 12 months post-injury [25]. After 4–5 weeks, these questions will be asked as part of the routine outpatient clinic visit.

#### Cost-effectiveness and work ability

A selection of the medical consumption questionnaire (iMCQ) and the productivity costs questionnaire (iCPQ) will be sent 1, 6 weeks, and 6 months post-injury. The questions which are not useful for the study, such as questions about occupational therapist appointments or a dietician appointment, were discarded. The general questions from both the questionnaires are the same, and these were combined into one questionnaire.

At the end of the study, 1 year post-injury, these questions will be asked again.

#### Patient-reported outcome

All of the following questionnaires will be sent 6 weeks and 3, 6, and 12 months post-injury.

- Patient-Rated Wrist Evaluation (PRWE) score [17]
- EQ-5D-5L [23]
- PROMIS Pain Interference (PROMIS PI)
- Visual analogue scale for pain and patient satisfaction (VAS)

#### Complications

Complications will be scored, 6 weeks and 3, 6, and 12 months post-injury.

The following will be scored:

- Secondary displacement
- McKay complication checklist [20]
- Budapest criteria for scoring CRPS [21]

#### Evaluation of implementing 1 week of plaster cast immobilization

After 1 year, when the inclusion period ends, all participating centers will be asked to fill out an evaluation form. This evaluation form will include questions such as if they prefer condition A or B, if condition B will be used as a new protocol in their hospital, feedback for the study, and changes for the protocol of condition A.

Figure 1 shows the time schedule for the used questionnaires.

### Time schedule

Inclusion of 440 patients will take place during a 1-year time period in the participating medical centers. A follow-up period of 12 months is taken into account and 6 months for data analysis and publication. See Fig. 1.

### Power analysis

We used the stepped wedge sample size tool in STATA.

A sample size of 330 patients was calculated with a power of 0.85, alfa of 0.05, and *ICC* of 0.01. We designed the stepped wedge for ten clusters with three patients per cluster per time period (Table 1). We used results from our pilot study for the reference values. The Patient-Rated Wrist Evaluation (PRWE) scores after 6 weeks were used as this will be one of our most important outcomes and have a validated clinical difference score. We used a PRWE score of 35 for the control group and 25 for the intervention group. A difference of around 10 points is needed to reach a relevant clinical difference [18]. In order to account for a 30% loss to follow-up, we aim for a sample size of 440 patients, i.e., four patients per cluster per time.

### Randomization

Each cluster will start with the usual care: condition A. The clusters/hospitals will be randomized for when to start with condition B. Patients are not randomized but follow the condition in their hospital. Castor will be used for randomizing the clusters. The clusters will be blinded only for the timing when they will start with the treatment of 1-week plaster cast immobilization: condition B. The researcher who will perform the randomization through castor will let the clusters/hospitals know when to start with condition B. At the beginning of the study, all hospitals will be informed about when they will switch from condition A to B. The data analysts will be blinded for the randomization of the clusters and when every cluster starts with condition B.

### Analysis of the material

All anonymized information gathered in the study will be stored in a Castor database at Radboud University Medical Center. The database is protected with passwords, and the data will be deleted 15 years after the study is officially closed. Only the research group will get full access to the database. Participating hospitals will get access to add data from their own participating patients. More information about the data management for the Cast OFF-2 study can be requested from the corresponding author on reasonable request.

### Statistical analysis

Descriptive analyses with median, range, and percentage will be used to describe demographic variables and outcomes such as age and gender. We will analyze the percentage of protocol violations and the willingness to participate from participating patients, hospitals, and physicians using a feedback evaluation. Patients will be asked to give feedback at the end of their follow-up period and physicians will be asked feedback after 6 months and 12 months.

A multivariate linear regression mixed model will be used to analyze the data from the different randomization groups and to account for clustering of repeated measurements within patients and within hospitals and time. We will analyze the effect of the intervention, in comparison with the usual care, on the PROs, return to activity, pain scores, and complication rate. We will adjust for confounding factors such as age, gender, and clinical parameters. The difference in complication rate will be determined using either a Fisher exact or a chi-square test, depending on the order of magnitude of the results. The difference in PCS-4 score, VAS score, and patient satisfaction will be determined using a *t*-test. The analysis will be performed by using the intention-to-treat model (according to the groups to which they were originally designed).

A cost-effective analysis will be performed additionally to the above-mentioned analyses. A budget impact analysis will be performed from various perspectives with the budgetary framework for the care as the base case perspective.

For missing data from patients who fulfilled the study, the available data will be analyzed if possible, for any interesting findings, and otherwise, the missing data will be imputed with replacement values using multiple imputation analysis.

### Ethics

The trial protocol and additional papers, including consent form, patient information sheet, and questionnaires, have been approved by the Dutch Ethical Review Board (METC) Arnhem-Nijmegen and have been declared exempt from the Medical research involving Human Subjects Act (WMO) (approval number: CMO 2021-7308).

### Oversight and monitoring of the study

Radboudumc will be the coordinating center for the Cast OFF-2 trial and will also provide the study coordinator. Every including hospital will have to provide a local research coordinator for their hospital. Every month the study coordinator will have contact with the participating centers to check if there are any problems and to discuss the inclusion rate per month (number of patients per month needed is four).

The Cast OFF-2 study has been declared exempt from the Medical research involving Human Subjects Act (WMO) and will therefore not have an interim analysis, auditing trial conduct, or data monitoring board. As this study was exempt from WMO, we do not need to officially report adverse events; however, we will screen for adverse events at 6 weeks and 3, 6, and 12 months post-injury. A list with information about the allocation and the hospitals will be securely saved. The head of the research team can provide this information if necessary for emergency need.

Relevant protocol amendments will be communicated to all participating hospitals, the funding party, and the ethical committee.

Trial results will be communicated to all participating hospitals and the funding party through a published article. A group authorship will be set up and used for the publication of the report of the Cast OFF-2. All authors should have contributed with the inclusions of the study and gathering the results and/or with writing the article. The research coordinators from the participating hospitals will be offered participation in the group authorship.

The Castor database will be used to safely store all information for the study. Castor automatically makes 4 times a day a backup of the data. All information per hospital is stored at a specific place on the local server which is protected by a password. The password is only known by the research group. All information will be stored for 15 years.

## Discussion

This publication presents an implementation study with the use of a randomized multicenter stepped wedge design for the use of 1 week of plaster cast immobilization after non-reduced DRF. This study will be used to implement 1 week of plaster cast immobilization in several hospitals. In addition, this study tries to answer the question whether 1 week of plaster cast gives lower PRWE scores, better patient satisfaction, earlier return to work, and lower costs and if it will have the same complication rate.

A limitation of this study is the variability in clinical judgment when to reduce a DRF and thereby the chance of bias due to the surgeon’s decision who expects a high chance of secondary displacement. However, the variability in deciding when to reduce a DRF is part of common daily practice. A second limitation may be the choice for a stepped wedge design instead of a randomized controlled trial. During the feasibility study, we encountered a lot of problems with patient cross-over, difficulty performing the study for the hospitals and a large number of patients who did not want to participate in the study. By using a stepped wedge design, we think the problems with the treatment protocol will be less variable. In addition, the greater variability in deciding when to reduce a DRF leads to the exclusion criteria which will be used in general practice. Therefore, this study will give an answer which can be directly translated into daily practice.

The strengths of this study are the pragmatic nature of this study, the fact that it is an implementation study (using the Dutch guideline) in combination with a comparative study, and the use of a cost-effectiveness analysis. The pragmatic nature of this study, using a stepped wedge design, leads to a very clear protocol to use for every hospital. A clear and easy protocol is necessary as this study is an implementation study. In addition, with this study design, we can include 440 patients in 1-year time. A cost-effectiveness analysis will be performed which will give extra evidence for the effectiveness of 1-week plaster cast immobilization. Other strengths of the study are the validated primary outcome measure (PRWE) and taking into account psychological views of post-traumatic pain using the PCS-4.

As this is an implementation study, we hope the results of this study will be used for the implementation of 1-week plaster cast immobilization for non-reduced DRF nationally.

## Trial status

The protocol was approved using version 2, date 15 January 2021. The recruitment of patients has not started. The expected start of recruitment will be in September 2021. A year later, the last recruitments of patients will be performed. The follow-up will finish 2 years after the start of the study.

## Data Availability

The datasets used and/or analyzed during the current study are available from the corresponding author on reasonable request.

## Abbreviations

ADL: Activities of daily living
CCMO: Central Committee on Research Involving Human Subjects; in Dutch: Centrale Commissie Mensgebonden Onderzoek
CRPS: Complex regional pain syndrome
CIRS: Cumulative Illness Rating Scale
DRF: Distal radius fracture
EQ-5D-5L: EuroQol-5 dimensions 5-level questionnaire
IC: Informed consent
iMCQ: Medical consumption questionnaire
METC: Medical research ethics committee (MREC); in Dutch: medisch ethische toetsing commissie (METC)
PCS-4: Pain Catastrophizing Scale-4
PRO: Patient-reported outcome
PROMIS: Patient-Reported Outcomes Measurement Information System
PROMIS PI: Patient-Reported Outcomes Measurement Pain Interference
PRWE: Patient-Reported Wrist Evaluation
iPCQ: Productivity costs questionnaire
QOL: Quality of life
RCT: Randomized clinical trial
VAS: Visual analogue scale
Wbp: Personal Data Protection Act (in Dutch: Wet Bescherming Persoonsgevens)
WMO: Medical Research Involving Human Subjects Act (in Dutch: Wet Medisch-wetenschappelijk Onderzoek met Mensen)
ZIN: Health care Institute of the Netherlands
